# Tuberculin Versus Physical Examination

**Published:** 1899-05

**Authors:** S. B. Willard

**Affiliations:** Yardley, Pa.


### TUBERCULIN *VERSUS* PHYSICAL EXAMINATION.

By S. B. Willard, V.S.,

YARDLEY, PA.

In December, 1898, I was called to examine a herd of ninety-six cows and heifers for tuberculosis. Among the herd was a grade Jersey cow which, while a good milker, was a very nervous animal, and gave a great deal of trouble to the milkers. The owner, having use for all his milk, isolated the cow, and made her nourish the other cows' calves; she had, I presume, raised some thirty calves in 1898. When I made the examination, in December, 1898, I examined twenty-four of them, and the reaction took place in thirteen (over 54 per cent.), also the cow which nursed them reacted. In March, 1899, Dr. F. Harker and myself examined part of the same herd, and eight calves which I considered too young in December, 1898, were examined, and four of them were condemned, all nourished by the same cow. I send you the result to show the danger of keeping tuberculous cows, and I think the above experience will show the fallacy of physical examination, as the above-mentioned cow was apparently very healthy.

